# A Comparison of Caregiver Burden for Different Types of Dementia: An 18-Month Retrospective Cohort Study

**DOI:** 10.3389/fpsyg.2021.798315

**Published:** 2022-01-17

**Authors:** Wen-Chien Huang, Ming-Che Chang, Wen-Fu Wang, Kai-Ming Jhang

**Affiliations:** ^1^Department of Neurology, Changhua Christian Hospital, Changhua, Taiwan; ^2^Department of Nuclear Medicine, Changhua Christian Hospital, Changhua, Taiwan; ^3^Department of Recreation and Holistic Wellness, Ming Dao University, Changhua, Taiwan

**Keywords:** dementia, caregiver burden, Lewy body disease, Zarit burden interview, neuropsychiatric symptoms

## Abstract

**Background:**

This study aimed to elucidate the influence of dementia etiologies on the degree of caregiver burden and determine which factors predict a high caregiving burden.

**Methods:**

This 18-month retrospective cohort study enrolled 630 patients and their caregivers from the Dementia Center of Changhua Christian Hospital. The care team performed face-to-face interviews every 6 months, for 18 months from when a diagnosis of dementia was made. The primary outcome was the change in Zarit Burden Interview (ZBI) scores. Generalized estimating equations were used for the longitudinal data analysis.

**Results:**

Participants with Lewy body disease (LBD) had a significantly higher caregiving burden compared with those with Alzheimer's disease (AD) (β = 3.83 ± 1.47, Wald = 6.79, *p* = 0.009) after adjusting for patient and caregiver features. Caregivers of mixed-type dementia and frontotemporal dementia (FTD) experienced a greater burden than caregivers of AD, at 6- and 18-month follow-up. Patients with more severe dementia, neuropsychiatric symptoms, being cared for by more than two caregivers, or utilizing social resources were associated with higher ZBI scores; the depressive mood of caregiver also predicted higher ZBI scores.

**Conclusion:**

This longitudinal study demonstrated that caregiver burden was influenced by the underlying dementia etiology of patients. The dementia care team should provide personalized education and transfer patients and caregivers to appropriate resources, especially for high-risk populations.

## Introduction

More than 40 million people around the world are living with dementia, and the prevalence is still increasing. Alzheimer's disease (AD) is the most common cause of dementia (Scheltens et al., 2016). Taking care of AD patients is difficult and is often associated with a high burden of care, especially over a prolonged period of time (van den Kieboom et al., 2020). The burden of caregiving can reduce the quality of life of caregivers and cause depressive symptoms (Liew et al., 2020). A high care burden typically also reduces the work productivity of caregivers (Fujihara et al., 2019).

Previous systemic reviews have revealed that factors associated with caregiver burden can be divided into three categories, namely, patient character, caregiver character, and family function (Chiao et al., 2015; van den Kieboom et al., 2020). Patient characteristics that increase caregiver burden include neuropsychiatric symptoms, daily functional limitations, and duration of illness. Caregiver features, such as poor physical and mental health status, low education level, cohabitation with the patient, and female gender, are associated with a higher burden of caregiving. Better family functioning and higher income have been reported to reduce the burden of the caregiver.

Most of the studies discussing dementia and caregiver burden are cross sectional (Huang et al., 2012; Yu et al., 2015; Hashimoto et al., 2017; Torrisi et al., 2017; Branger et al., 2018; Kawano et al., 2020; Lucijanić et al., 2020; Jhang et al., 2021; Tsai et al., 2021). Most of these articles supported that patient neuropsychiatric symptoms (Huang et al., 2012; Hashimoto et al., 2017; Torrisi et al., 2017; Branger et al., 2018; Kawano et al., 2020; Tsai et al., 2021), poor performance of activities for daily living (ADLs) (Hashimoto et al., 2017; Kawano et al., 2020; Tsai et al., 2021), duration of disease (Kawano et al., 2020), female gender, and low education level of the caregiver (Jhang et al., 2021) increased the caregiving burden. Worse cognitive function (Yu et al., 2015), using daily care services, and the male sex of the patient (Lucijanić et al., 2020) were also shown to increase the care burden in some cross-sectional articles.

There are relatively few longitudinal studies focusing on the burden of caring for people living with dementia (Shim et al., 2016; Chen et al., 2017; Pillemer et al., 2018; Ku et al., 2019; Connors et al., 2020). The trajectory of caregiver burden is highly variable in different studies (van den Kieboom et al., 2020), but factors predicting a high burden of care are similar across both cross-sectional and longitudinal studies, including the presence of neuropsychiatric symptoms (Shim et al., 2016; Chen et al., 2017; Ku et al., 2019; Connors et al., 2020), poor ADL function (Connors et al., 2020), and female caregiver (Pillemer et al., 2018). Inability to drive (Connors et al., 2020) and anosognosia of the patient (Perales et al., 2016) have also been reported to increase the burden of care.

Several regional studies have compared the burden of caregivers between different types of dementia (Yeager et al., 2010; D'Onofrio et al., 2015; Oliveira et al., 2015; Branger et al., 2018; Liu et al., 2018; Kawano et al., 2020), and the results of these studies were obscure. Two Asian studies conducted in Japan and China mentioned that caregivers taking care of subjects with Lewy body dementia (LBD) or frontotemporal dementia (FTD) experienced more burden than those caring for patients with AD dementia (Liu et al., 2018; Kawano et al., 2020). Another study reported that vascular dementia caregivers in Italy had a lower care burden than AD caregivers due to the higher rate of female caregivers and the longer length of time spent caring for AD patients (D'Onofrio et al., 2015). However, some reports did not observe any differences in caregiver burden between dementia subtypes (Yeager et al., 2010; Oliveira et al., 2015; Branger et al., 2018). Branger et al. (2018) reported that caregiver burden was similar irrespective of dementia etiology, including AD, FTD, and vascular dementia. Oliveira et al. (2015) reported that caregivers of LBD and AD dementia have no statistical difference in caregiving burden. Yeager et al. (2010) also concluded there were no differences in the burden of care between vascular dementia and AD dementia.

Determining factors that predict the burden of caregiving is vital for addressing care needs for both people living with dementia and their care partners (Jhang et al., 2019). However, previous studies have presented inconsistent results regarding the association between dementia subtype and caregiver burden.

To the best of our knowledge, there has not been a study discussing the trajectory of caregiver burden in different types of dementia. Longitudinal studies, including multiple dementia subtypes, are scarce. This study used retrospective cohort data to compare the burden from caregiving of several subtypes of dementia diagnosed by a subspecialist. Most of the common neurodegenerative dementia, vascular cognitive impairment (VCI), and dementia with mixed AD and vascular contribution were analyzed. This study aimed to elucidate factors associated with the burden of care and determine the long-term trajectory of caregiver burden in different types of dementia.

## Methods

### Participants

This 18-month retrospective cohort study was conducted at the Dementia Center of Changhua Christian Hospital (CCH), a medical center in Central Taiwan. In October 2015, the hospital established dementia collaborative multidisciplinary care model for supporting community-residential patients with dementia and their care partners. Team members included physicians (e.g., neurologists, psychiatrists, gerontologists, and primary care physicians), psychologists, social workers, dieticians, occupational therapists, pharmacists, and nursing case managers. The care team performed face-to-face interviews every 6 months from when the patients were newly diagnosed with dementia. The interviews assessed the cognitive function, living status, behavioral and psychological symptoms of the patient, and the care burden and mood of the caregiver. All data were recorded in electronic charts by the nursing case managers. The care team stopped following up if the patients did not visit the dementia clinic for more than 6 months, refused the assessment, became nursing home residents, or expired. People living with dementia and their caregivers who were assessed between October 2015 and November 2020 were included in the analysis. The inclusion criteria of participants were as follows: (1) the patients who met the diagnosis of dementia and (2) the patients and their caregivers who agreed to participate at least once in a face-to-face interview, in which the results were recorded in the electronic chart. The patients and their caregivers were excluded if they did not receive interviews during October 2015 and November 2020.

Neurologists and psychiatric specialists made the dementia diagnosis through clinical interviews and biomarkers, including neuropsychological tests and brain images [magnetic resonance imaging (MRI), positron emission tomography (PET), TRODAT, and amyloid PET]. The National Institute on Aging-Alzheimer's Association (NIA-AA) (Albert et al., 2011; McKhann et al., 2011), the International Society for Vascular Behavioral and Cognitive Disorders (VASCOG) (Sachdev et al., 2014), the Movement Disorder Society Task Force criteria (Emre et al., 2007), the Fourth consensus report of dementia with Lewy bodies (DLBs) Consortium (McKeith et al., 2017), and the International consensus criteria for behavioral variant FTD (Rascovsky et al., 2011) were used for the diagnosis of AD, VCI, Parkinson's disease dementia (PDD), DLBs, and FTD, respectively. Patients who fit both the NIA-AA criteria for AD and the VASCOG criteria for possible major vascular cognitive disorder were classified as having mixed dementia. LBD included subjects with a diagnosis of PDD or DLB.

This study was approved by the Institutional Review Board (IRB) of CCH (CCH IRB 201218). Since all data needed in this study were extracted from electronic charts after the deletion of personalized information, the need for informed consent was waived by the IRB of CCH.

### Measurements

#### The Measurement of Patient Features

The characteristics of patients such as gender, age, education level, underlying medical illness, and dementia subtype were collected at the initial assessment. Reports of getting lost/disorientated, utilization of an allowance or resources, marital and cohabitation status, and ambulatory condition were assessed and recorded at each interview. The Clinical Dementia Rating Scale Sum of Box (CDR-SOB) was used to determine the severity of dementia (Morris, 1993). The severity of dementia-related psychological and behavioral symptoms was assessed using the Neuropsychiatric Inventory (NPI). The use of resources was classified into five groups: (1) not using any social resources, (2) day care centers, (3) community stations (community dementia care centers or community elderly stations), (4) home services (resident care attendant providing bathing or household chores at home), and (5) both services (use home and community services simultaneously). ADL was categorized as dependent if any one of the activities needed assistance from others.

#### Measurement of Caregiver Factors

The caregiver factors such as age, education level, marital status, and relation to the patient were recorded at each interview. The Zarit Burden Interview (ZBI) instrument and Center for Epidemiologic Studies Depression (CES-D) Scale were used to assess the burden and depressive mood of the caregiver (Lewinsohn et al., 1997; Bédard et al., 2001). The primary caregiver was the carer who answered the ZBI/CES-D and was one of the primary care providers. There were five care modes in the study: Mode 0 (the ADL of patient was independent and the caregiver only accompanied the patient), Mode 1 (care by a sole informal caregiver), Mode 2 (care by more than two caregivers, which could include a foreign care worker), Mode 3 (care at different children homes alternately), and Mode 4 (care by a sole foreign care worker).

### Statistical Analyses

All data were analyzed using R software (R Foundation for Statistical Computing). Pearson's chi-square test or Fisher's exact test was used to assess the differences in categorical data. Numerical data were tested using Student's *t*-test or Kruskal-Wallis rank-sum test. Generalized estimating equations (GEEs) and the Wald χ^2^ statistic in conjunction with a first-order autoregressive working correlation matrix (AR1) were applied for the longitudinal data analysis. The model used time as an impact factor and allowed us to observe the disparity degree of ZBI between groups more precisely. Differences were considered statistically significant when *p*-value was < 0.05.

## Results

### Characteristics of Patients and Caregivers at Baseline and Follow-Up

A total of 630 pairs of patients and caregivers were enrolled in this study at baseline. Notably, 201, 121, and 76 patient and caregiver dyads were completed at the 6-, 12-, and 18-month follow-up, respectively. Table 1 shows the patient and caregiver characteristics between baseline and follow-up. Most of the patient characteristics do not show a significant difference between baseline and follow-up, except for CDR-SOB and the percentage of resource utilization. The CDR-SOB score increased gradually during follow-up. The utilization rate of social resources increased from 16.5% at baseline to 30.3% at 18-month follow-up.

**Table 1:** Characteristics of patients and caregivers at baseline and follow-up.

**Characteristic**	**Baseline**	**6 months**	**12 months**	**18 months**	***P*-value**
	***N* = 630**	***N* = 201**	***N* = 121**	***N* = 76**	
**Patient factors**					
Male (%)	230 (36.5%)	70 (34.8%)	37 (30.6%)	24 (31.6%)	0.561
Age (%)					0.981
<70	75 (11.9%)	27(13.4%)	12 (9.9%)	9 (11.8%)	
70–80	231 (36.7%)	72(35.8%)	46 (38.0%)	26 (34.2%)	
>80	324 (51.4%)	102(50.7%)	63 (52.1%)	41 (53.9%)	
CDR-SOB (SD)	6.10 (4.68)	7.03 (5.14)	7.02 (4.77)	7.20 (4.50)	**0.019**
NPI (SD)	13.02 (57.30)	15.90 (71.32)	19.52 (91.02)	11.09 (13.11)	0.704
Education years (SD)	4.91 (4.40)	5.21 (4.53)	5.24 (4.40)	5.29 (4.57)	0.723
Ambulation (%)					0.448
Free	417 (66.2%)	135(67.2%)	72 (59.5%)	54 (71.1%)	
Assisted device	151 (24.0%)	51(25.4%)	41 (33.9%)	18 (23.7%)	
Wheelchair	60 (9.5%)	15 (7.5%)	8 (6.6%)	4 (5.3%)	
Bedridden	2 (0.3%)				
Diagnosis (%)					0.883
AD	369 (58.6%)	121 (60.2%)	58 (47.9%)	44 (57.9%)	
VCI	106 (16.8%)	30(14.9%)	28 (23.1%)	13 (17.1%)	
Mixed^¥^	19 (3.0%)	5 (2.5%)	3 (2.5%)	1 (1.3%)	
LBD	61 (9.7%)	20 (10.0%)	11 (9.1%)	8 (10.5%)	
FTD	16 (2.5%)	5 (2.5%)	5 (4.1%)	2 (2.6%)	
Others	59 (9.4%)	20 (10.0%)	16 (13.2%)	8 (10.5%)	
**Disease (%)**					
DM	176 (27.9%)	30 (14.9%)	71 (58.7%)	24 (31.6%)	0.660
HTN	354 (56.2%)	107 (53.2%)	71 (58.7%)	39 (51.3%)	0.666
Dyslipidemia	225 (35.7%)	64 (31.8%)	42 (34.7%)	22 (28.9%)	0.558
CKD	60 (9.5%)	16 (8.0%)	12 (9.9%)	8 (10.5%)	0.885
CVD	56 (8.9%)	18 (9.0%)	12 (9.9%)	4 (5.3%)	0.708
CVA	77 (12.2%)	27 (13.4%)	19 (15.7%)	4 (5.3%)	0.171
ADL dependent (%)	233 (37.0%)	85 (42.3%)	49 (40.5%)	29 (38.2%)	0.565
Widow or not-married (%)	262 (41.6%)	80 (39.8%)	49 (40.5%)	29 (38.2%)	0.926
Cohabitation (%)					0.912
Live alone	33 (5.2%)	14 (4.1%)	3 (2.5%)	2 (2.6%)	
Spouse only	134 (21.3%)	75 (22.1%)	29 (24.0%)	14 (18.4%)	
Spouse/children	196 (31.1%)	99 (29.1%)	38 (31.4%)	29 (38.2%)	
Children only	213 (33.8%)	125 (36.8%)	40 (33.1%)	24 (31.6%)	
Others	54 (8.6%)	27 (7.9%)	11 (9.1%)	7 (9.2%)	
Allowance (%)					0.269
No	192 (30.5%)	53 (26.4%)	24 (19.8%)	24 (31.6%)	
Government insurance	404 (64.1%)	134 (66.7%)	88 (72.7%)	24 (31.6%)	
Labor pension	34 (5.4%)	14 (7.0%)	9 (7.4%)	6 (7.9%)	
Resources^#^ (%)					**0.013**
No use	526 (83.5%)	153 (76.1%)	88 (72.7%)	53 (69.7%)	
Day care center	31 (4.9%)	14 (7.0%)	8 (6.6%)	6 (7.9%)	
Community station	20 (3.2%)	15 (7.5%)	13 (10.7%)	7 (9.2%)	
Home service	9(7.8%)	16 (8.0%)	10 (8.3%)	8 (10.5%)	
Both services	4 (0.6%)	3 (1.5%)	2 (1.7%)	2 (2.6%)	
Getting lost (%)	191 (30.3%)	60 (29.9%)	35 (28.9%)	19 (25.0%)	0.812
**Caregiver factors**					
ZBI (SD)	28.08 (16.79)	27.58 (17.68)	26.82 (15.20)	27.84 (17.02)	0.892
CES-D (SD)	13.28 (10.40)	12.26 (10.30)	11.88 (9.61)	13.32 (10.98)	0.407
Carer age (SD)	57.92 (13.70)	58.63 (12.96)	58.53 (12.28)	57.57 (13.45)	0.879
Carer Education years (SD)	12.57 (11.02)	13.35 (12.87)	13.20 (11.91)	11.88 (3.68)	0.705
ZBI relationship (%)					0.895
Spouse	111 (17.6%)	42 (20.9%)	24 (19.8%)	14 (18.4%)	
Children	369 (58.6%)	118 (58.7%)	70 (57.9%)	47 (61.8%)	
Others	150 (23.8%)	41 (20.4%)	27 (22.3%)	15 (19.7%)	
Primary caregiver (%)	627 (99.5%)	200 (99.5%)	119 (98.3%)	75 (98.7%)	0.452
Widow or not-married (%)	108 (17.1%)	37 (18.4%)	16 (13.2%)	9 (11.8%)	0.415
Care mode^¶^(%)					0.091
Mode 0	92 (14.6%)	24 (11.9%)	16 (13.2%)	6 (7.9%)	
Mode 1	232 (36.8%)	74 (36.8%)	46 (38.0%)	22 (28.9%)	
Mode 2	222 (35.2%)	75 (37.3%)	34 (28.1%)	31 (40.8%)	
Mode 3	11 (1.7%)	4 (2.0%)	0(0%)	1 (1.3%)	
Mode 4	73 (11.6%)	24 (11.9%)	25 (20.7%)	16 (21.1%)	

*CDR-SOB, clinical dementia rating scale-sum of boxes; NPI, Neuropsychiatric Inventory; AD, Alzheimer's disease; VCI, vascular cognitive impairment; LBD, Lewy body disease; FTD, frontotemporal dementia; DM, diabetes mellitus; HTN, hypertension; CKD, chronic kidney disease; CVD, cardiovascular disease; CVA, cerebrovascular disease; CES-D, The Center for Epidemiologic Studies Depression Scale; ZBI, Zarit Burden Interview.*

*^¥^Mixed type indicates mixed AD and VCI.*

*^#^Resources indicate the utilization status of social resources when ZBI was recorded. “Community stations” include community dementia care centers or community elderly stations. “Home services” indicate bathing or household chores provided by a resident care attendant. “Both services” indicate the subject used home and community services simultaneously.*

*^¶^Care mode indicates how caregivers take care of people living with dementia most of the time. Mode 0 = ADL independent, caregivers only accompanied the subject; Mode 1 = cared for by a solo informal caregiver; Mode 2 = cared for by more than two caregivers (can include foreign care worker); Mode 3 = care at different children home alternately; Mode 4 = care by a solo foreign care worker.*

*The bold values are highlighted as the results with statistical significance, indicated P-values <0.05*.

The caregiver factors such as age, years of education, marital status, care mode, ZBI, and CES-D scores showed no significant differences between baseline and follow-up. The age of caregivers at baseline was 57.92 ± 13.7 years, and more than 98% of caregiver data was collected from the primary caregiver.

### Comparison of the Characteristics Between Follow-Up and Lost-To-Follow-Up Groups

A total of 244 patient and caregiver dyads were completed at least once during follow-up, and 89 patient/caregiver pairs did not reach the first follow-up (6-month) during the study period. A comparison of the characteristics between the follow-up and lost-to-follow-up groups is shown in Table 2. The lost-to-follow-up group used significantly fewer social resources (12.8 vs. 21.3%, *p* = 0.008) and were significantly more likely to be cared for by other relatives (not spouse or children, 29 vs. 18.4%, *p* = 0.018). The CDR-SOB and other factors, including age, years of education, marital status, the severity of dementia, neuropsychiatric behavior symptoms, ambulation status, comorbidities, ADL dependency, dementia subtype, care mode, caregiver burden, or depressed mood, showed no significant differences between the two groups.

**Table 2:** Comparison of baseline characteristics of patients and caregivers between the follow-up and lost-to-follow-up groups.

**Characteristic**	**Followed up at**	**Lost to follow up**	***P*-values**
	**least one time**	***N* = 297**	
	***N* = 244**		
**Patient factors**			
Male (%)	94 (38.5%)	95 (32.0%)	0.135
Age (%)			0.140
<70	25 (10.2%)	38 (12.8%)	
70–80	102 (41.8%)	100 (33.7%)	
>80	117 (48.0%)	159 (53.5%)	
CDR-SOB (SD)	5.96 (4.61)	6.16 (4.56)	0.622
NPI (SD)	18.76 (90.45)	9.71 (13.39)	0.089
Education years (SD)	5.15 (4.45)	4.75 (4.50)	0.297
Ambulation (%)			0.333
Free	154(63.1%)	202 (68.0%)	
Assisted device	68(27.9%)	69 (23.2%)	
Wheelchair	22(9.0%)	24 (8.1%)	
Bedridden	0 (0.0%)	2 (0.7%)	
Diagnosis (%)			0.178
AD	131(53.7%)	182 (61.3%)	
VCI	45(18.4%)	48 (16.2%)	
Mixed^¥^	6(2.5%)	5 (2.5%)	
LBD	25(10.2%)	8 (2.7%)	
FTD	6(2.5%)	7 (2.4%)	
Others	31 (12.7%)	19 (6.4%)	
**Disease (%)**			
DM	71 (29.1%)	77 (25.9%)	0.467
HTN	134 (54.9%)	161 (54.2%)	0.938
Dyslipidemia	88 (36.1%)	97 (32.7%)	0.459
CKD	25 (10.2%)	25 (8.4%)	0.561
CVD	16 (6.6%)	29 (9.8%)	0.235
CVA	25 (10.2%)	37 (12.5%)	0.504
ADL dependent (%)	90 (36.9%)	117 (39.4%)	0.611
Widow or not-married (%)	93 (38.1%)	139 (46.8%)	0.052
Cohabitation (%)			0.335
Live alone	9(3.7%)	18 (6.1%)	
Spouse only	55(22.5%)	63 (21.2%)	
Spouse/children	80(32.8%)	80 (26.9%)	
Children only	81(33.2%)	104 (35.0%)	
Others	19 (7.8%)	32 (10.8%)	
Allowance (%)			0.766
No	80(32.8%)	91 (30.6%)	
Government insurance	152 (62.3%)	188 (63.3%)	
Labor pension	12 (4.9%)	18 (6.1%)	
Resources^#^ (%)			**0.008**
No use	192(78.7%)	259 (87.2%)	
Day care center	11(4.5%)	17(5.7%)	
Community station	13 (5.3%)	4 (1.3%)	
Home service	25 (10.2%)	16 (5.4%)	
Both services	3 (1.2%)	1 (0.3%)	
Getting lost	66 (27.0%)	94 (31.6%)	0.284
**Caregiver factors**			
ZBI (SD)	28.30 (16.28)	28.51 (17.15)	0.885
CES-D (SD)	13.32 (10.76)	12.99 (9.97)	0.713
Carer age (SD)	57.92 (13.70)	58.28 (13.92)	0.725
Carer education years (SD)	12.00 (8.13)	13.09 (13.08)	0.254
ZBI_Relationship (%)			**0.018**
Spouse	48(19.7%)	50 (16.8%)	
Children	151(61.9%)	161 (54.2%)	
Others	45 (18.4%)	86 (29.0%)	
Primary caregiver (%)	243 (99.6%)	295 (99.3%)	1.000
Widow or not-married (%)	39 (16.0%)	47 (15.8%)	1.000
Care mode^¶^ (%)			0.857
Mode 0	35(14.3%)	37 (12.5%)	
Mode 1	86(35.2%)	118 (39.7%)	
Mode 2	88(36.1%)	103 (34.7%)	
Mode 3	4(1.6%)	5 (1.7%)	
Mode 4	31 (12.7%)	34 (11.4%)	

*^¥^Mixed type indicates mixed AD and VCI.*

*^#^Resources indicate the utilization status of social resources when ZBI was recorded. “Community stations” include community dementia care centers or community elderly stations. “Home services” indicate bathing or household chores provided by a resident care attendant. “Both services” indicate the subject used home and community services simultaneously.*

*^¶^Care mode indicates how caregivers take care of people living with dementia most of the time. Mode 0 = ADL independent, caregivers only accompanied the subject; Mode 1 = cared for by a solo informal caregiver; Mode 2 = cared for by more than two caregivers (can include foreign care worker); Mode 3 = care at different children home alternately; Mode 4 = care by a solo foreign care worker.*

*The bold values are highlighted as the results with statistical significance, indicated P-values <0.05*.

### Factors Associated With Caregiving Burden

GEEs were applied to determine which factors were associated with high ZBI scores (Table 3). The following patient factors were significantly associated with caregiving burden: CDR-SOB and neuropsychiatric symptoms, a history of cardiovascular disease (CVD), resource utilization, cohabitation with children, and a diagnosis of LBD. The characteristics of patients such as gender, age, education level, ambulatory status, ADL dependency, marital status, the presence of getting lost, and allowance utilization were not significantly associated with caregiving burden.

**Table 3:** Generalized estimating equations to determine factors associated with caregiver burden.

	**ß (SE)**	**Wald**	***p*-value**
(Intercept)	2.93 (5.38)	0.30	0.586
**Patient factors**			
Male	−0.65 (1.00)	0.41	0.520
Age (Ref: <70)			
70–80	0.06 (1.7)	0.00	0.972
>80	0.30 (1.8)	0.03	0.868
CDR-SOB	0.38 (0.13)	8.99	**0.003**
NPI	0.01 (<0.01)	7.63	**0.006**
Education (years)	0.16 (0.12)	1.66	0.198
**Ambulation (ref: free)**			
Assisted device	0.25 (1.02)	0.06	0.804
Wheelchair	−0.17 (1.52)	0.01	0.912
Bedridden	1.60 (5.22)	0.09	0.759
**Diagnosis (ref: AD)**			
VCI	−0.07(1.30)	0.00	0.958
Mixed^¥^	3.79 (2.68)	1.99	0.158
LBD	3.83 (1.47)	6.79	**0.009**
FTD	0.99 (2.69)	0.14	0.712
Others	1.61 (1.42)	1.28	0.258
**Disease**			
DM	−0.48 (0.90)	0.29	0.590
HTN	0.09 (0.83)	0.01	0.914
Dyslipidemia	1.15 (0.86)	1.79	0.181
CKD	0.05 (1.43)	0.00	0.980
CVD	−3.07 (1.27)	5.82	**0.016**
CVA	−0.32 (1.37)	0.05	0.818
ADL dependent	0.11 (1.143)	0.01	0.921
Widow or not-married	2.02 (1.75)	1.33	0.250
**Cohabitation (ref: live alone)**			
Spouse only	−1.38 (2.27)	0.37	0.543
Spouse/children	−1.93 (2.18)	0.78	0.376
Children only	−3.24 (1.60)	4.13	**0.042**
Others	−2.69 (2.05)	1.73	0.190
**Allowance (Ref: no)**			
Government insurance	−0.16 (0.95)	0.03	0.868
Labor pension	3.45 (1.94)	3.16	0.075
**Resources**^**#**^ **(ref: no use)**			
Day care center	2.30 (1.95)	1.39	0.238
Community station	2.68 (1.65)	2.65	0.104
Home service	4.27 (1.40)	9.25	**0.002**
Both services	7.98 (3.06)	6.77	**0.009**
Getting lost	0.53 (0.91)	0.34	0.561
**ZBI followed time (ref: baseline)**			
6 months	−0.18 (0.99)	0.03	0.854
12 months	−0.37 (1.16)	0.10	0.748
18 months	−1.29 (1.31)	0.98	0.322
**Caregiver factors**			
CES-D	1.02 (0.04)	525.65	**<0.001**
Carer age	−0.08 (0.04)	3.54	0.060
Carer education (years)	0.05 (0.04)	1.46	0.227
**ZBI_Relationship (ref: spouse)**			
Children	−0.56 (1.45)	0.15	0.698
Others	−0.28 (1.66)	0.03	0.868
Primary caregiver	11.05 (3.32)	11.08	**<0.001**
Widow or not-married	−1.39 (1.18)	1.40	0.237
**Care mode (ref: 0)** ^**¶**^			
Mode 1	1.47 (1.31)	1.26	0.262
Mode 2	2.82 (1.33)	4.49	**0.034**
Mode 3	5.88 (3.54)	2.75	0.097
Mode 4	0.67 (1.82)	0.13	0.715

*^¥^Mixed type indicates mixed AD and VCI.*

*^#^Resources indicate the utilization status of social resources when ZBI was recorded. “Community stations” include community dementia care centers or community elderly stations. “Home services” indicate bathing or household chores provided by a resident care attendant. “Both services” indicate the subject used home and community services simultaneously.*

*^¶^Care mode indicates how caregivers take care of people living with dementia most of the time. Mode 0 = ADL independent, caregivers only accompanied the subject; Mode 1 = cared for by a solo informal caregiver; Mode 2 = cared for by more than two caregivers (can include foreign care worker); Mode 3 = care at different children home alternately; Mode 4 = care by a solo foreign care worker.*

*The bold values are highlighted as the results with statistical significance, indicated P-values < 0.05*.

#### Patient Factors Associated With Caregiving Burden

CDR-SOB, NPI, a diagnosis of LBD, and using home services were associated with higher caregiver burden. CDR-SOB and NPI showed a positive relationship with ZBI score (estimate = 0.38, SE = 0.13, Wald = 8.99, *p* = 0.003 and estimate = 0.013, SE = <0.01, Wald = 7.63, *p* = 0.006, respectively). Participants diagnosed with LBD were associated with higher ZBI scores (estimate = 3.83, SE = 1.47, Wald = 6.79, *p*=0.009). Patients using home services (estimate = 4.27, SE = 1.40, Wald = 9.25, *p* = 0.002) or both services were associated with higher caregiver burden (estimate = 8.00, SE = 3.06, Wald = 6.77, *p* = 0.009).

Patients with a history of CVD had lower ZBI scores (estimate = −0.32, SE = 1.37, Wald = 5.82, *p* = 0.016). Patients living with children had lower burden scores compared with those living alone (estimate = −3.24, SE = 1.60, Wald = 4.13, *p* = 0.042).

#### Caregiver Factors Associated With Lower Caregiving Burden

Caregiver factors related to the ZBI score included the mood of carer, care mode, and if the ZBI responder was the primary caregiver. The CES-D score of the caregiver was significantly associated with a higher ZBI (estimate = 0.98, SE = 0.06, Wald = 307.56, *p* < 0.001). Being the primary caregiver was also associated with a higher caregiving burden (estimate = 12.05, SE = 5.90, Wald = 4.14, *p* = 0.042). Patients who were cared for by more than two caregivers had increased ZBI scores compared with patients who only needed accompanying (estimate = 2.28, SE = 1.33, Wald = 4.49, *p* = 0.034).

### Dementia Diagnosis and Caregiving Burden at Different Follow-Up Periods

**Figure 1:**
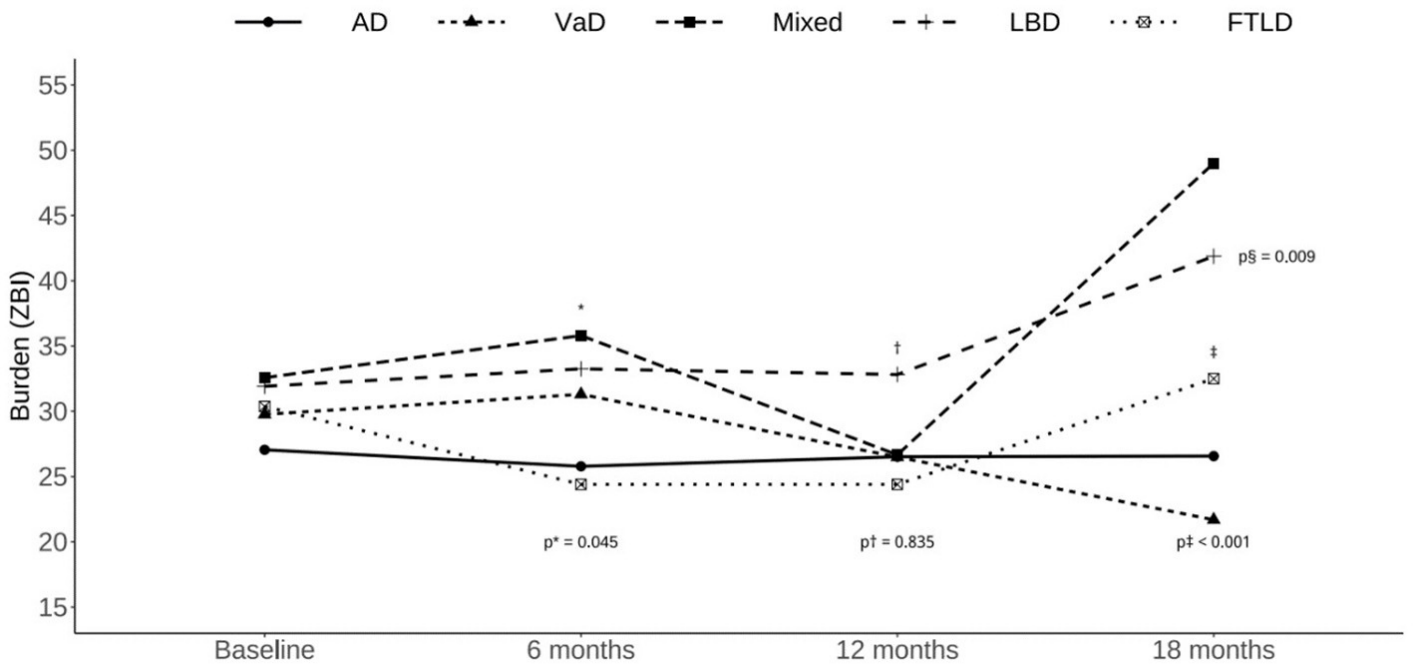
The trajectory of Zarit Burden Interview scores in different dementia subtypes.

Figure 1 shows the mean ZBI score of GEE model according to the follow-up time and dementia subtype. The face-to-face interview was held on months 6, 12, and 18 after enrolling in this study. Patients and caregivers who completed the 6-month follow-up showed significantly higher ZBI scores for patients diagnosed with mixed-type dementia compared with AD type dementia (estimate = 11.59, SE = 5.77, Wald = 4.03, *p* = 0.045). A total of 201 patient and caregiver dyads completed the first interview at 6-month follow-up. Also, 89 patients and caregivers did not reach the first follow-up during the study period (6 months). All baseline characteristics were not significantly different between the complete follow-up and no follow-up groups (*n* = 340).

Participants who completed the 12-month follow-up showed significantly higher ZBI scores in subjects diagnosed with LBD compared with those diagnosed with AD (estimate = 7.81, SE = 3.07, Wald = 6.47, *p* = 0.011). A total of 121 patients and caregivers finished the 12-month follow-up, while 146 patients did not reach the 12-month follow-up time in the study period. Also, 363 patient and caregiver dyads were lost-to-follow-up. Lost-to-follow-up patients had a significantly higher rate of AD diagnosis (61.2 vs. 47.9%, *p* = 0.044) and tended to be cared for by more than two caregivers (38.8 vs. 26.4%, *p* = 0.017).

The 18-month complete follow-up group showed significantly higher ZBI scores in subjects diagnosed with FTD compared with those diagnosed with AD (estimate = 22.16, SE = 5.09, Wald = 18.95, *p* < 0.001). A total of 76 patients and caregivers completed the 18-month follow-up evaluation. There were 257 patients and caregivers who did not reach the 18-month follow-up time in the study period. Also, 297 patients were lost-to-follow-up. The complete follow-up group showed significantly higher NPI scores (33.75 vs. 9.71, *p* = 0.011) and a higher percentage used social resources (19.7 vs. 12.8%, *p* = 0.034) than the lost-to-follow-up group.

## Discussion

This study found that the dementia subtype influenced the degree of caregiving burden. Caregivers taking care of patients with LBD had a significantly increased burden compared with those taking care of patients with AD. Kawano et al. (2020) and Liu et al. (2018) concluded that caregivers of DLB patients have a higher burden than those of AD patients, which may be due to the increased rate of neuropsychiatric symptoms observed in DLB patients. Although not statistically significant, the mean baseline NPI score was higher in LBD patients compared with the AD population in this study (13.6 vs. 11.7, *p* = 0.71). Other possible explanations included the significantly higher risk of fall and ADL dependency in the LBD group (47.5 vs. 31.4%, *p* < 0.001). Past literature found that the caregiver burden was more affected by depression and motor features in the LBD group, but not by cognitive decline (de Oliveira et al., 2020). Independent ADL function has been reported to be a risk factor associated with the burden of care (Hashimoto et al., 2017; Kawano et al., 2020; Tsai et al., 2021). Oliveira et al. (2015) found there was no difference in caregiver burden in the LBD and AD subjects (ZBI = 16.79 vs. 18.59, *p* = 0.42) in a cross-sectional study. This discrepancy may be associated with different study designs, the heterogeneity, and ethnic difference among cases included. More studies are needed to clarify the caregiver burden between LBD and AD subjects.

Caregivers of FTD patients revealed a higher care burden compared with caregivers of AD patients at the 18-month follow-up in this study. This same finding has been previously reported by Liu et al. (2018). The authors suggest that the increased burden is due to the higher rate of behavioral and psychiatric symptoms observed in FTD.

In this study, patients with mixed dementia were associated with an increased caregiver burden than AD patients at the 6-month follow-up. Branger et al. (2018) compared the ZBI scores of AD, vascular dementia, and mixed dementia caregivers and showed that the care burden was similar irrespective of the dementia etiology of patient. Overlap of different neuropathologies could lead to faster disease progression (Matej et al., 2019), which may be associated with a higher caregiving burden.

This study revealed that higher CDR-SOB and NPI scores predicted higher caregiving burden; these findings were consistent with previous studies (Huang et al., 2012; Shim et al., 2016; Chen et al., 2017; Hashimoto et al., 2017; Torrisi et al., 2017; Branger et al., 2018; Ku et al., 2019; Connors et al., 2020; Kawano et al., 2020; Tsai et al., 2021).

Caregivers taking care of patients with CVD comorbidity had a lower burden. The reason for this is not apparent but could be related to the intensive medical control of chronic disorders in CVD populations before the dementia diagnosis was confirmed. This causal relationship needs to be further investigated and clarified. Cohabitation with children was associated with lower ZBI scores compared with patients living alone. Viñas-Diez et al. (2017) had a similar result and showed that patient cohabitation with children reduced caregiver burden. Participants using home services or home and community services were associated with a high caregiver burden. However, this result is controversial. Connors et al. (2020) reported that patients using home service have a lower caregiver burden than patients living at home without the service. In contrast, Wang et al. (2021) found that patients with home services were more ADL dependent and associated with a higher caregiving burden, thus resulting in caregivers utilizing care resources. The presented results are consistent with the latter study and support the theory that utilization of supplementary health care resources is an indirect indicator for higher ADL dependency of the patients.

The depressive mood of caregiver, primary caregiver, and care by more than two caregivers were all associated with a higher burden of care. van den Kieboom et al. (2020) and Chiao et al. (2015) reported that the mental status of caregivers was associated with caregiver burden. More than two caregivers care for patients who are cared for may have more complex care needs, leading to a higher caregiver burden.

A strength of this study is the integrity of the dementia subtypes. Few previous studies have compared the caregiver burden between different etiological diagnoses (Branger et al., 2018; Liu et al., 2018; Kawano et al., 2020). This study compared the association between five types of dementia and caregiver burden. A range of potential patient and caregiver factors associated with the degree of caregiving burden were also included.

Besides, there were several study limitations. First, only 45.1% of patients completed at least one follow-up record. However, a comparison of the characteristics between complete follow-up and lost-to-follow-up groups showed no significant differences. Second, detailed ADL scores, such as the Barthel Index, were missing. ADL function was categorized as a dependent if any of the activities were assisted or reminded by others. This dichotomy may explain the non-significant association between ADL dependency and caregiver burden in this study. Third, the number of study participants was fewer in FTD and LBD groups than AD and vascular dementia groups. The relatively small sample size of the two groups influenced the representativeness of this study. Fourth, because of the retrospective cohort design, some valuable confounding factors were not available. Management of neuropsychiatric symptoms such as acetylcholinesterase inhibitor or behavioral therapy was lacking, interfering with the caregiver burden.

## Conclusion

This retrospective cohort study demonstrated that the etiology of underlying dementia influenced caregiver burden. Caregivers taking care of patients diagnosed with LBD experienced a higher care burden than those taking care of patients with AD. The cognitive function and neuropsychiatric symptoms of patients and the mood and care mode of caregivers were also associated with the degree of caregiver burden. The dementia collaborative care team may provide appropriate education and support resources, especially for the high-risk population.

## Data Availability Statement

The original contributions presented in the study are included in the article/supplementary material, further inquiries can be directed to the corresponding author/s.

## Ethics Statement

The studies involving human participants were reviewed and approved by the Institutional Review Board of Changhua Christian Hospital (CCH IRB 201218). Written informed consent was not required to participate in this study in accordance with the local legislation and institutional requirements.

## Author Contributions

K-MJ, M-CC, and W-FW designed the study. M-CC analyzed the data. K-MJ and W-CH wrote the manuscript. All authors contributed to the article and approved the submitted version.

## Funding

This study was supported by a grant from Changhua Christian Hospital (Grant No. 110-CCH-IRP-037).

## Conflict of Interest

The authors declare that the research was conducted in the absence of any commercial or financial relationships that could be construed as a potential conflict of interest.

## Publisher's Note

All claims expressed in this article are solely those of the authors and do not necessarily represent those of their affiliated organizations, or those of the publisher, the editors and the reviewers. Any product that may be evaluated in this article, or claim that may be made by its manufacturer, is not guaranteed or endorsed by the publisher.
