# Corrigendum: A comparison of caregiver burden for different types of dementia: an 18-month retrospective cohort study

**DOI:** 10.3389/fpsyg.2023.1224716

**Published:** 2023-06-13

**Authors:** Wen-Chien Huang, Ming-Che Chang, Wen-Fu Wang, Kai-Ming Jhang

**Affiliations:** ^1^Department of Neurology, Changhua Christian Hospital, Changhua, Taiwan; ^2^Department of Nuclear Medicine, Changhua Christian Hospital, Changhua, Taiwan; ^3^Department of Recreation and Holistic Wellness, Ming Dao University, Changhua, Taiwan

**Keywords:** dementia, caregiver burden, Lewy body disease, Zarit burden interview, neuropsychiatric symptoms

In the published article, there was an error in the Ethics statement as published. The Ethics statement incorrectly stated that written informed consent was obtained for participation in the study and for the publication of any potentially identifiable images or data included in the article. However, written informed consent was not required for participation in this study in accordance with the local legislation and institutional requirements, and no potentially identifiable human images/data were included in the published article. The updated Ethics statement is included below:

## Ethics statement

The studies involving human participants were reviewed and approved by the Institutional Review Board of Changhua Christian Hospital (CCH IRB 201218). Written informed consent was not required to participate in this study in accordance with the local legislation and institutional requirements.

The authors apologize for this error and state that this does not change the scientific conclusions of the article in any way. The original article has been updated.

